# miR-200 family promotes podocyte differentiation through repression of RSAD2

**DOI:** 10.1038/srep27105

**Published:** 2016-06-02

**Authors:** Zhigui Li, Hongqiang Yin, Shuang Hao, Lifeng Wang, Jing Gao, Xiaoyue Tan, Zhuo Yang

**Affiliations:** 1 College of Medicine, State Key Laboratory of Medicinal Chemical Biology, Tianjin Key Laboratory of Tumor Microenvironment and Neurovascular Regulation, Nankai University, Tianjin 300071, China

## Abstract

Mature podocytes are highly differentiated cells with several characteristic phenotypic features that are involved in the glomerular filtration function. During kidney development, a series of changes of the morphological characteristics and cellular functions may happen in podocytes. The miR-200 family functions in various biological and pathological processes. But the underlying molecular mechanisms of miR-200 family that functions in podocyte differentiation remain poorly understood. Herein is shown that miR-200a, miR-200b and miR-429 are significantly upregulated during the differentiation of podocytes, with highest upregulation of miR-200a. In these cells, restraint of miR-200 family by RNA interference assay revealed a prominent inhibition of cell differentiation. More intriguingly, miR-200 family directly inhibited the radical S-adenosyl methionine domain-containing protein 2 (RASD2) expression. Moreover, further upregulation of RSAD2 combining with restraint of miR-200 family revealed a promotion of podocyte dedifferentiation and proliferation. In addition, the expression of RSAD2 is consistent with that of *in vitro* podocyte differentiation in prenatal and postnatal mouse kidney, and significantly down-regulated during the kidney development. Together, these findings indicate that miR-200 family may potentially promote podocyte differentiation through repression of RSAD2 expression. Our data also demonstrate a novel role of the antiviral protein RSAD2 as a regulator in cell differentiation.

The mature podocytes, also known as glomerular epithelial cells, are highly differentiated cells that reside on the glomerular basement membrane (GBM). During glomerulogenesis, podocytes develop from precursor cells, which arise from induced mesenchymal renal stem cells, into their adult phenotype, which is characterized by a complex pattern of processes^1^. The function of podocytes is mainly based on their special structure and includes regulation of the glomerular filter. Recently, several studies have indicated that the ectopic development of podocytes may lead to abnormal glomerulogenesis and subsequent kidney diseases. Kidneys of *Lmx1b* mutant mice exhibit pathological changes, which places podocytes at the center of the pathomechanism leading to proteinuria, hematuria, and chronic renal disease^2^,^3^. Concordantly, The recent discovery of several novel podocyte proteins and their mutation analysis, including the Nephrin homologue Neph1^4^, Nephrin^5^, CD2-associated protein(CD2AP)^6^, Podocin^7^, and transient receptor potential cation channel 6 (TRPC6)^8^,^9^, have indicated the critical role of the structural integrity of podocytes in maintaining the normal function of the glomerular filtration barrier.

MicroRNAs (miRNAs) are single-stranded, noncoding RNA molecules that are thought to modulate gene expression by translational inhibition and destabilization of messenger RNAs (mRNAs)^10^,^11^. Since the first miRNA, the *lin-4*, was found in the nematode worm *Caenorhabditis elegans*^12^, miRNAs are present in multiple species, including plants, animals and even an algae^13^, implying that the regulation of genes by RNA silencing is an ancient mechanism. In fact, some new emerging studies have reported that podocyte-specific deletion of Dicer or Drosha result in proteinuria and glomerulosclerosis, which suggests the important role of miRNAs in podocytes for renal function.

The miR-200 family of miRNAs comprises five members organized as two clusters, miRs-200b/a/429 and miRs-200c/141, on chromosomes 1 and 12 in humans and 4 and 6 in mice. Although abundant studies of the role of miR-200 family in the oncology field have been accumulated, the contribution of the family in nephrogenesis, especially in specific cell types during differentiation, is still largely unknown. Raman Agrawal *et al*., showed that miR-200 family was one of the most abundant miRNAs in kidney^14^. miR-200 was expressed in the developing pronephros, the skin and part of the somites^15^. However, the accurate role of miR-200 family in podocyte differentiation is not clear. Therefore, a study in our laboratory has identified the miRNAs and mRNA expression profiles in undifferentiated and differentiated podocytes using miRNA and mRNA microarray^16^. These works lay the groundwork for our further molecular mechanism study in podocyte differentiation.

RSAD2, also known as Viperin or Cig5, plays a key role in the innate immune response system. RSAD2 is rapidly induced by both type I and II interferons and has a wide variety of antiviral activity^17^. RSAD2 subsequently localizes to the endoplasmic reticulum (ER) via its N-terminal amphipathic α-helix^18^, the process of which appears to result in the disruption of lipid raft microdomains and the prevention of influenza virus from budding of the plasma membrane^19^. Although increasing amounts of researchers have reported on the antiviral effect of RSAD2 in various organs and cells, very few studies have been conducted on the expression or function of system during cell development. One recent study from Steinbusch *et al*. has shown that RSAD2 knockdown induces ER stress and skews the chondrocyte phenotype towards hypertrophy in developing chondrocytes *in vivo*^20^. However, whether and how the RSAD2 is regulated by miRNA, especially by miR200 family during podocyte differentiation is still unclear.

Herein, we hypothesize that miR-200 family promotes podocyte differentiation through repression of RSAD2 expression. Our results indicate that the expression of miR-200 family is significantly up-regulated in differentiated podocytes comparing with the undifferentiated one, and the inhibition of miR-200 family can arrest cell differentiation. Furthermore, bioinformatic analysis and Luciferase reporter assay revealed that RASD2 was directly negatively regulated by miR-200 family. Together, we show that miR-200 family and RASD2 cooperate to assure a normal differentiation of podocytes.

## Results

### Upregulation of miR-200a, miR-200b and miR-429 during the differentiation of podocytes

**Figure 1:**
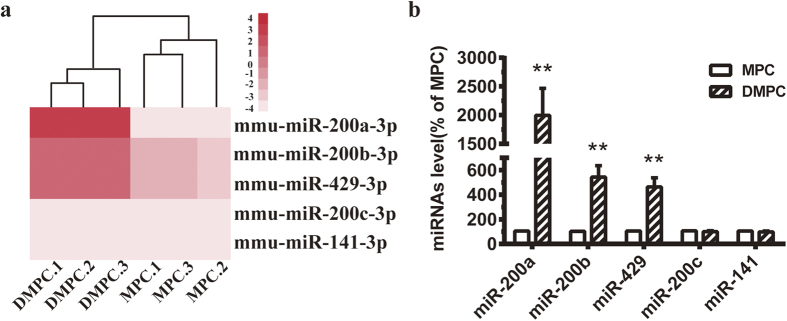
Upregulation of miR-200a, miR-200b and miR-429 during the differentiation of podocytes. (**a**) Our previous research of heat map of differentially expressed miRNA profiling in MPCs and DMPCs^16^. Differential profiling using microarrays included three samples per group. Orangered indicates upregulated and light pink shows downregulated miRNAs. The right number of the map ‘−4 to 4’ represents the z-value range. The bottom labels from DMPC.1 to MPC.2 represent sample names of different groups. (**b**) Validation of the expression of miR-200a, miR-200b, miR-429, miR-200c and miR-141 by miRNA-specific qPCR. Triplicate assays were done for each RNA sample and the relative amount of each miRNA was normalized to GAPDH. The expression of miRNAs in MPC groups was standardized as percentile change of numerical one hundred. The data are the average of three independent experiments and are shown as the mean ± S.D. Statistically significant differences between two groups was indicated by **P < 0.01.

To investigate the expression level of miR-200 family during the differentiation of podocytes, a recent study in our laboratory has identified the miRNAs and mRNA expression levels in undifferentiated and differentiated podocytes using miRNA and mRNA microarray^16^. The miRNA microarray showed that miR-200a, miR-200b and miR-429 expressions were significantly increased in differentiated podocytes (Fig. 1a). Notably, the three members are all located on chromosome 4, which indicated a potential key role of the three members of miR-200 family in podocyte differentiation. Further real-time quantitative PCR (qPCR) assay showed that miR-200a (1996 ± 470, *P* < 0.01), miR-200b (543 ± 92, *P* < 0.01) and miR-429 (463 ± 74, *P* < 0.01) expressions were significantly increased in differentiated podocytes (Fig. 1b).

### Restraint of miR-200 family revealed inhibition of cell differentiation and apoptosis

**Figure 2:**
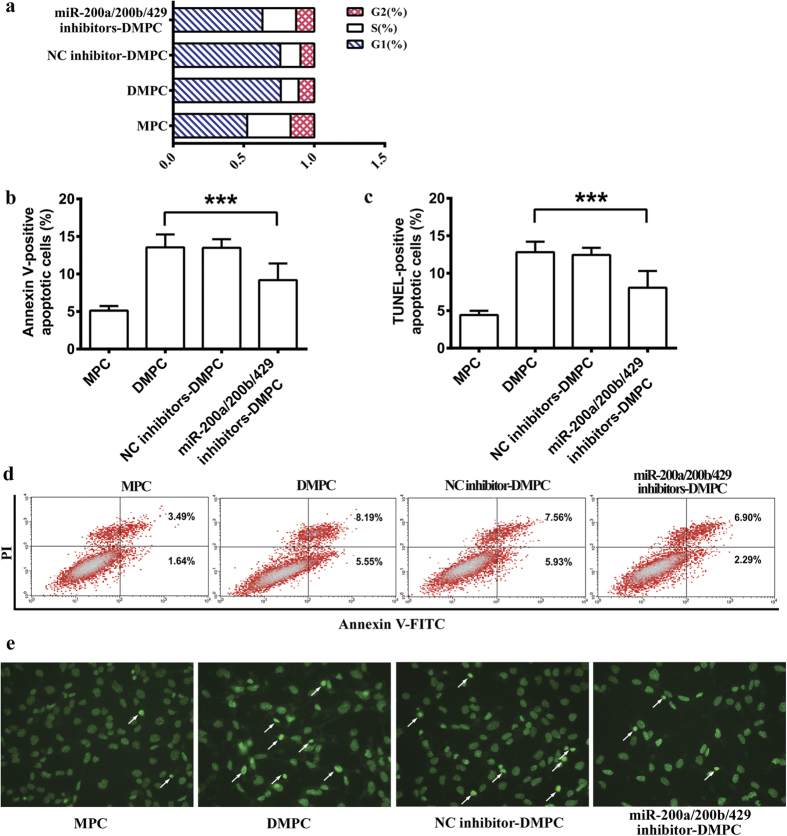
Restraint of miR-200 family affects cell cycle and apoptosis. The DMPCs were transfected with microRNA inhibitor negative control or combination of miR-200a/200b/429 inhibitors and then grouped as NC inhibitor, miR-200a/200b/429 inhibitors-DMPC, DMPC, and MPC, respectively. (**a**) Cell-cycle distribution of podocytes were detected by a flow cytometer. (**d**) Cells transfected as in (**a**) were suspended and subjected to Annexin V-FITC/PI analysis. Dot plots show intensity of Annexin V-FITC fluorescence on the X-axis and PI fluorescence on the Y-axis. Lower left quadrant (LL): living cells (Annexin V^−^/PI^−^), Upper left quadrant (UL): necrotic cells (AnnexinV^−^/PI^+^), Upper right quadrant (UR): late apoptotic cells and necrotic cells (Annexin V^+^/PI^+^), Lower right quadrant (LR): early apoptotic cells (Annexin V^+^/PI^−^). Statistical result of Annexin V-positive apoptotic cell rate (LR + UR) was shown in (**b**). (**e**) Morphological validation of cell apoptosis was detected by TUNEL assay. The arrows showed the apoptotic cells (fluorescent-FITC, green). The intact figure showed a costaining with DAPI in the supplementary data (Fig. S1). Statistical result of TUNEL-positive apoptotic cell rate was shown in (**c**). The data are the average of three independent experiments and are shown as the mean ± S.D. Statistically significant differences between two groups was indicated by ***P < 0.001.

To confirm the biological function of miR-200 family in podocyte differentiation, we examined cell cycle distribution after a simultaneous inhibition of the expression of miR-200a, miR-200b and miR-429. Compared with DMPC group (76.3 ± 0.5%), a significant decrease of cells in the G0/G1 phase of cell cycle was observed following miR-200a, miR-200b and miR-429 co-inhibition (miR-200a/200b/429 inhibitors-DMPC group; 63.3 ± 4.5%, *P* < 0.001)(Fig. 2a).

**Figure 3:**
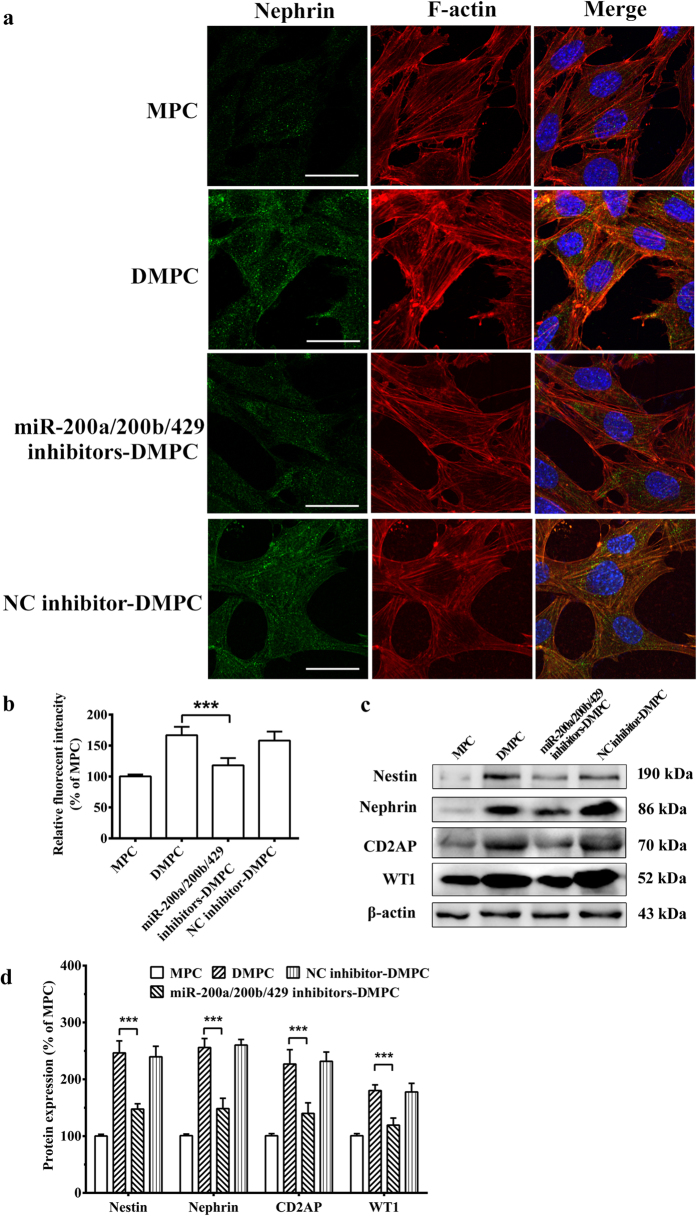
Restraint of miR-200 family affects differentiation marker and reveals rearrangement of cytoskeleton. (**a**) Cells transfected as in Fig. 2 were detected by immunofluorescence assay. Nephrin (green) and F-actin staining (red) of podocytes transfected with a microRNA inhibitor negative control or combination of miR-200a/200b/429 inhibitors were detected at 48 h. DAPI staining was used to detect nuclei and is merged with Nephrin and F-actin in their respective panels. The scale bar represents 20 μm. (**b**) Semiquantification of F-actin expression. The mean relative influorecent intensity in each cell of the four groups was analyzed. (**c**) Nestin, Nephrin, CD2AP and WT1 expression were detected by western blot assay. The cropped blots were shown, and the corresponding full-length blots were shown in the supplementary data (Figures S2–S4). (**d**) Corresponding histogram of Nestin, Nephrin, CD2AP and WT1 expression in western blot assay. The data are the average of three independent experiments and are shown as the mean ± S.D. Statistically significant differences between two groups are indicated by ***P < 0.001.

In addition, research has revealed a rearrangement of cytoskeleton during differentiation of MPCs^1^,^21^. An ordered array of actin fibers and microtubules extends into the forming cellular processes during differentiation. These cytoskeletal rearrangements and process formation are accompanied by the onset of Nephrin, CD2AP and WT1 synthesis, the process-associated proteins marking specifically differentiated podocytes. Consequently, we studied the effect of miR-200 family inhibition on the rearrangement of cytoskeleton and expression of biomarkers, which would reveal the effect of miR-200 family on podocyte differentiation. Indeed, comparative analysis of F-actin distribution revealed that the process extension was prominently inhibited in miR-200a/200b/429 inhibitors-DMPC (Fig. 3a). Semiquantification of F-actin expression showed that, compared with the DPMC group (166.7 ± 13.6%), the mean relative fluorescent intensity in each cell of the miR-200a/200b/429 inhibitors-DPMC group is significantly decreased (118.7 ± 11.6%, *P* < 0.001, Fig. 3b). Besides, Nestin, the intermediate filaments-associated protein, was significantly down-regulated in miR-200a/200b/429 inhibitors-DMPC group (147.6 ± 9.3%) compared with the DMPC group (246.4 ± 21.1%, *P* < 0.001, Fig. 3c,d). Moreover, The expressions of podocyte-specific markers, Nephrin, CD2AP and WT1 significantly down-regulated in miR-200a/200b/429 inhibitors-DMPC group (148.3 ± 18.1% of Nephrin, *P* < 0.001; 139.8 ± 18.6% of CD2AP, *P* < 0.001; 119.2 ± 12.5% of WT1, *P* < 0.001, Fig. 3c,d) compared with the DMPC group (256.0 ± 15.8% of Nephrin, *P* < 0.001; 226.8 ± 25.3% of CD2AP, *P* < 0.001; 180.2 ± 9.9% of WT1, *P* < 0.001, Fig. 3c,d).

Since restraint of miR-200 family inhibited podocyte differentiation, we further explored the effect of restraint of miR-200 family on podocyte apoptosis. Indeed, the TUNEL assay showed that restraint of miR-200a, miR200b and miR-429 evidently decreased the apoptotic cell numbers (Fig. 2c,e). Subsequent flow cytometry analysis showed that restraint of miR-200a, miR200b and miR-429 significantly decreased the rate of Annexin V-positive apoptotic cells in DMPC group (9.19 ± 2.2%) compared with the DMPC group (13.56 ± 1.7%, *P* < 0.001, Fig. 2b,d).

### RSAD2 was a predicted target gene of miR-200 family

**Figure 4:**
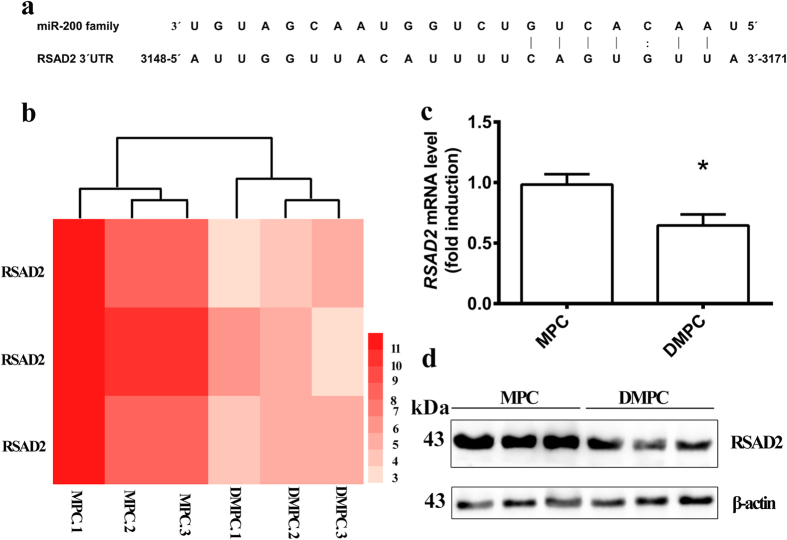
RSAD2 is a predicted target gene of miR-200 family. (**a**) Sequence of putative miR-200 family binding sites in the 3′-UTR of mouse RSAD2. (**b**) Our previous research of heat map of RSAD2expression in MPCs and DMPCs^16^. Differential profiling using microarrays included three samples per group. Red indicates upregulated and light pink shows downregulated RSAD2. The right number of the map ‘3 to 11’ represents the z-value range. The bottom labels from MPC.1 to DMPC.3 represent sample names of different groups. (**c**)Validation of the expression of *RSAD2*mRNA. (**d**)Validation of the expression of RSAD2 protein. A cropped blot was shown, and the corresponding full-length blot was shown in the supplementary data (Fig. S5). Data are the average of three independent experiments and are shown as the mean ± S.D. Statistically significant differences between two groups was indicated by *P < 0.05.

We then looked for miR-200 family putative targets. On the basis of previous data, In general, the predicted miR-200 family target genes were downregulated in DMPCs because miR-200 family expression was upregulated in DMPCs. Then, we further predicted the miR-200 family target genes by comparing the upregulated miRNAs and the downregulated mRNAs on two web sites: http://www.microrna.org/ microrna/getGeneForm.do and http://mirdb.org/miRDB/. Both of the web sites showed the same sequences of *RSAD2* targeting by miR-200 family (Fig. 4a). In accordance with our hypothesis, our previous mRNA microarray study showed a significant down-regulation of *RSAD2* in DMPCs (Fig. 4b)^16^. Therefore, we confirmed a prominent down-regulation of RSAD2 mRNA (*P* < 0.05, Fig. 4c) and protein expression in DMPCs (*P* < 0.01, Fig. 4d).

**Figure 5:**
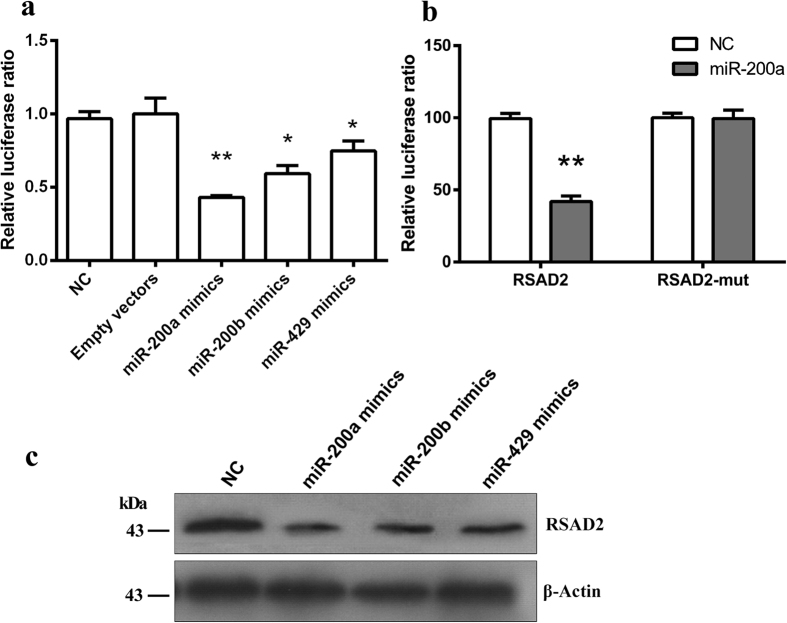
The miR-200 family directly regulates RSAD2 expression by targeting the 3′-UTR of RSAD2. Luciferase reporter assays of wild type (WT) and mutated (MUT) 3′-UTR sequences of RSAD2 in the presence of miR-200a, miR-200b, miR-429 mimics or negative control. (**a**) Luciferase activity was analyzed after co-transfection with miR-200a, miR-200b, miR-429 mimics or the negative control with the psiCHECK-WT-*RSAD2* wild-type plasmid. (**b**) Luciferase activity was analyzed after co-transfection with miR-200a mimic or the negative control with the psiCHECK-WT-*RSAD2* wild-type plasmid or mutant plasmid (psiCHECK-MT-*RSAD2*) in HEK293 cells. (**c**) TheRSAD2 protein levels were examined using Western blot analysis. Quantification of the bands was performed using the Image J software, and β-actin was used as an internal control. The data are the average of three independent experiments and are shown as the mean ± S.D. Statistically significant differences are indicated (*P < 0.05, **P < 0.01).

To investigate whether miR-200 family can indeed target RSAD2, a dual luciferase reporter assay was conducted. The miR-200a, miR-200b and miR-429 mimics and vectors containing the 3′-UTR of *RSAD2* were transiently transfected into HEK293 cells. To test the *RSAD2* putative binding site, we generated a mutant *RSAD2* construct, *RSAD2*-mut, in which the 3′-UTR of *RSAD2* was altered using a site-directed mutagenesis kit. Results showed that miR-200a (*P* < 0.01), miR-200b (*P* < 0.05) and miR-429 (*P* < 0.05) significantly down-regulated the luciferase activity of *RSAD2* construct (Fig. 5a), whereas luciferase activity was not generated from the mutant construct (Fig. 5b). In addition, Western blot assay showed that miR-200 family (miR-200a, miR-200b and miR-429) evidently affected protein levels of RSAD2 (Fig. 5c). All together, these results suggested that miR-200 family directly regulated the expression ofRSAD2 by interacting with its 3′-UTR.

### Over-expression of RSAD2 combining with restraint of miR-200 family revealed promotion of cell dedifferentiation and proliferation

**Figure 6:**
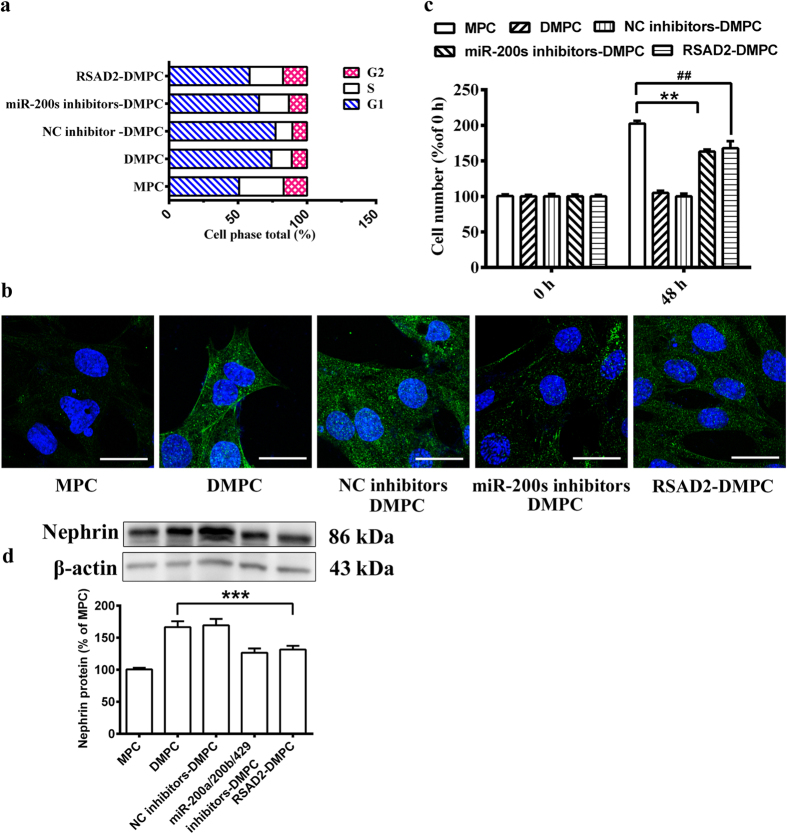
Over-expression of RSAD2 combining with restraint of miR-200 family revealed inhibition of cell differentiation and proliferation. The DMPCs were co-transfected microRNA inhibitor negative control or combination of miR-200a/200b/429 inhibitors with pEGFP-*RSAD2* plasmid and then grouped as NC Inhibitor, miR-200s Inhibitors + RSAD-DMPC, miR-200s Inhibitors-DMPC, DMPC, and MPC, respectively. (**a**) Cell-cycle distribution of the podocytes were detected by a flow cytometer. (**b**) Nephrin (green) of podocytes was detected after 48 h. DAPI staining was used to detect nuclei and is merged with Nephrin in their respective panels. The scale bar represents 20 μm. (**c**) Cell proliferation were analysed by WST-8 assay at 48 h. (**d**) Corresponding histogram of Nephrin protein expression in western blot assay. A cropped blot was shown above the histogram, and the corresponding full-length blot was shown in the supplementary data (Fig. S6). Data are means ± S.D. Statistically significant differences are indicated (**P < 0.01, ^##^*P* < 0.01).

As shown above, overexpression of miR-200 family inhibited RSAD2 in HEK293 cells. If this also holds true for podocytes, one might predict that restraint of miR-200 family expression combining with higher expression of RSAD2 may restrict podocyte differentiation. Relative to miR-200 family inhibition cells (miR-200s inhibitors DMPC group; 65.2 ± 8.9%), a decrease cells in G0/G1 phase of the cell cycle was observed following miR-200 family inhibition combining with overexpression of RSAD2 in DMPC (RSAD2-DMPC group; 58.4 ± 1.7%, *P* < 0.05) (Fig. 6a). Similarly, Nephrin, a biomarker of podocyte differentiation, was down-regulated after the same treatment in DMPC, which indicated a restriction of podocyte differentiation (Fig. 6b). Western blot assay showed an accordant significant downregulation of Nephrin in RSAD2-DMPC group (131.50 ± 5.9%) compared with the DMPC group (166.62 ± 9.2%, *P* < 0.001, Fig. 6d). In addition, cell proliferation was analyzed by WST-8 assay. Cell proliferation in RSAD2-DMPC group (167.7 ± 9.9%), was partially increased compared with the miR-200s inhibitors DMPC group in 48 h (163.1 ± 2.8%, *P* < 0.01, Fig. 6c). Together, functional assays in podocytes demonstrated that miR-200 family inhibited RSAD2 to promote podocyte differentiation.

### The expression of RSAD2 in mouse renal cortex

**Figure 7:**
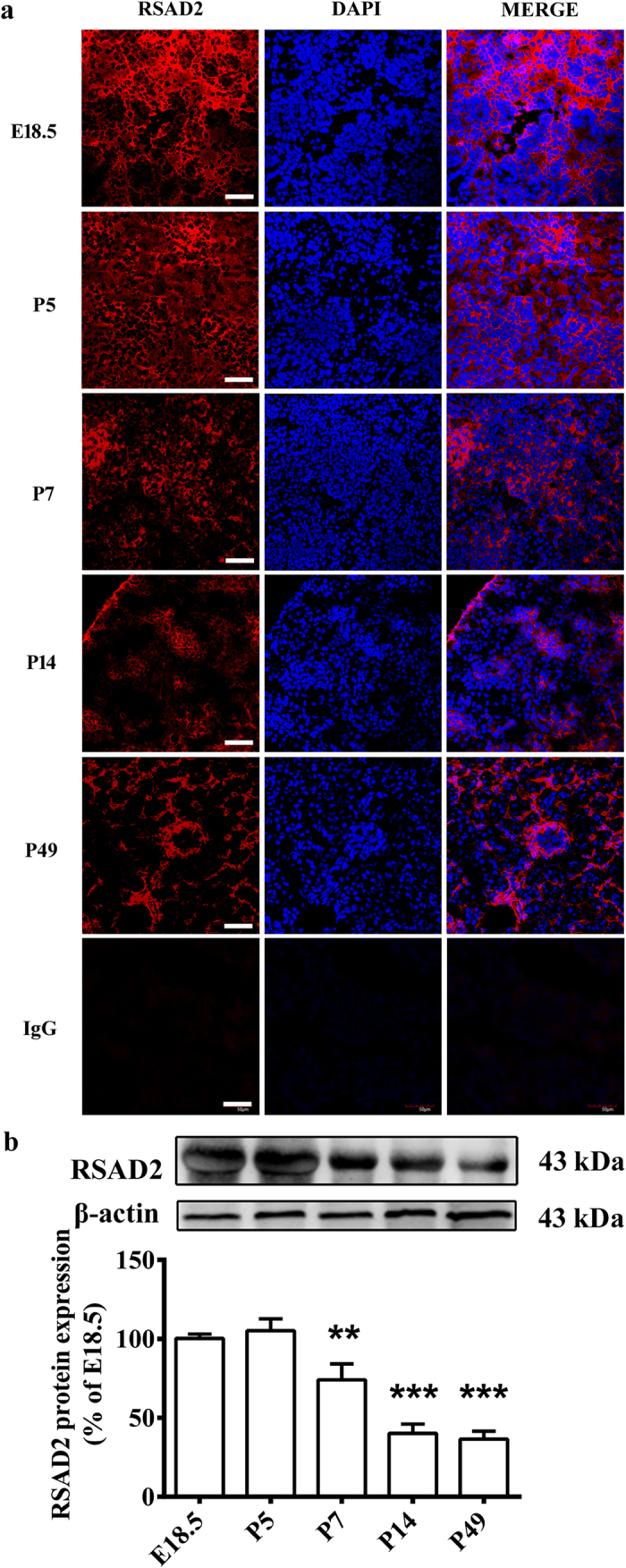
The expression of RSAD2 in mouse renal cortex. RSAD2 expression was examined in mouse renal cortex at E18.5, P5, P7, P14 and P49. (**a**) Immunofluorescence staining was used to examine the RSAD2 expression. The negative control image showed the renal cortex stained with a species-appropriate IgG. Scale bar, 50 μm. (**b**) Western blot assay was used to quantitatively evaluate the RSAD2 expression. A cropped blot was shown above the histogram, and the corresponding full-length blot was shown in the supplementary data (Fig. S7). Data are means ± S.D. Statistically significant differences are indicated (**P < 0.01, ***P < 0.001).

Next, we analyzed the appearance of RSAD2 in developing renal cortex by examining its expression in the prenatal and postnatal mouse kidney that displays glomeruli at different stages of development. We used immunofluorescence assay to detect RSAD2 expression in the mouse kidney at prenatal day (E) 18.5 and postnatal day (P) 5, 7, 14 and 49. As shown in Fig. 7a, RSAD2 expression was found at all stages of renal glomerulus and tubule formation during prenatal and postnatal development. Importantly, the expression of RSAD2 was major localized in the glomerulus, but not the tubule at P14 and P49. The result indicates a positively correlative of RSAD2 with podocyte during its differentiation. Moreover, there was a decrease in the expression of RSAD2 in glomeruli between P5 and P7. Further analysis of RSAD2 expression in renal cortex was conducted by using western blot assay. Consistently, compared with the E18.5 group, the expression of RSAD2 significantly down-regulated at P7 (*P* < 0.01, Fig. 7b), P14 (*P* < 0.001, Fig. 7b) and P49 (*P* < 0.001, Fig. 7b). These results are consistent with results *in vitro* study in podocytes.

## Discussion

In the present study, we show a relationship between miR-200 family and RSAD2 in podocyte differentiation. The results showed that miR-200 family modulated cell differentiation through the inhibition of RSAD2. Furthermore, the blockade of miR-200 family by RNA interference inhibited the expression of RASD2 and arrested cellular differentiation. Our studies suggested the hypothetic miR-200s/RSAD2 signal is crucial for podocyte differentiation. Since functional defects of differentiating podocytes may lead to structural damage of glomerulus and failure of the glomerular filtration barrier, which in turn triggers a cascade of events that can finally lead to renal disease. Thus, our findings emphasize that the miR-200s/RSAD2 signal may represent a promising pathway in exploring molecule mechanisms of podocyte differentiation.

Several studies have indicated that miRNAs play crucial roles in kidney development. The first functional studies addressing roles for miRNAs in podocytes in kidney have used a conditional approach to knock down Dicer, which is required for the production of mature miRNAs^22^,^23^,^24^.

Our recent study also indicated the miRNA-mRNA regulatory networks during podocyte differentiation^16^. In this study, we found that miR-200a, miR-200b and miR-429 were upregulated in DMPCs, whereas the expression of miR-200c and miR-141 were not affected (Fig. 1). Notably, the three miR-200a/200b/429 are all located on chromosome 4, which indicates a potential key role of the three members on chromosome 4 in podocyte differentiation, but not the other two members (miR-200c and miR-141) on chromosome 6. Thus, a negative control or miR-200a, miR-200b and miR-429 inhibitors were all co-transfected together into DMPCs for 48 h. We found that restraint of miR-200 family significantly inhibited podocyte differentiation (Figs 2 and 3). Though the members of the miR-200 family (the miR-200b/200c/429 group and the miR-200a/141 group) share the similarities of their seed sequences^25^, the biological function of miR-200a/200b/429 subfamily in our study may undergo a chromosome-dependent molecular mechanism.

As regulators of gene expression, miRNAs can work by either targeting the mRNA for destruction or by inhibiting protein synthesis^26^,^27^. Because miR-200a/200b/429 were up-regulated in DMPCs, we examined the down-regulated genes in DMPCs to predict the target genes using the microRNA and miRDB databases. Finally, we selected the *RSAD2* gene as a putative target gene. The RSAD2 (Viperin/Cig5) is known to be an antiviral protein^17^,^28^. However, the RSAD2 is recently reported to be a novel chondrogenic regulator in developing chondrocytes *in vivo*^20^.

We have reported a downregulation of *RSAD2* in DMPCs using a mRNA microarray study (Fig. 4b)^16^. We further confirmed a prominent down-regulation of RSAD2 protein expression in DMPCs (Fig. 4c).We then constructed a vector containing the mutated 3′-UTR of the *RSAD2* gene, and confirmed that the *RSAD2* gene was regulated by miR-200a/200b/429 subfamily using a dual luciferase reporter assay (Fig. 5). These data indicated that miR-200a/200b/429 negatively regulated RSAD2 expression by binding to the 3′-UTR of the *RSAD2* gene.

Several genes have been reported to be the targets of the miR-200 family, including ZEB1^29^, ZEB2^30^, Foxf2^31^ and SIP1^32^. However, most of them were reported to be involved in cancer. Our further assay by restraint of miR-200 family expression combining with higher expression of RSAD2 in podocytes showed a restriction of podocyte differentiation (Fig. 6), which further confirmed the pivotal role of miR-200s/RSAD2 signaling in podocyte differentiation. Besides, we also revealed the role of miR-200s/RSAD2 signaling in podocyte proliferation. Inhibition of the miR-200s/RSAD2 signaling significantly increased the podocyte proliferative rate (Fig. 6). Since report has revealed the critical role of mir-200 family in cell proliferation^33^, These results indicated that the novel role of the miR-200s/RSAD2 signaling in podocyte proliferation.

The RSAD2 is generally considered as an antiviral protein. The RSAD2 would be induced by interferons to resist the infection of virus^34^. We initially doubted the RSAD2 signaling may result from the treatment of interferon in podocyte culture. However, in our study, the RSAD2 maintained at a low level in interferon-induced MPCs (Fig. 4b,c), which indicated an entirely different role of RSAD2 may play in podocytes. One recent study from Steinbusch *et al*. have shown a novel role of the RSAD2 in developing chondrocytes *in vivo*^20^. Here we bring direct new evidences that miR-200s-mediated RSAD2 plays a crucial role in podocyte differentiation.

We should point out that signals controlling podocyte differentiation may have cross-talk with each other, we cannot exclude the possibility that other signals may participate in the miR-200s/RSAD2 signal. Therefore, further study is needed to investigate a clear signaling network that may take part in the miR-200s/RSAD2 signal. In that regard, the anti-viral action of RSAD2 by localizing to the endoplasmic reticulum^28^, may be a new potential direction in miR-200s-mediated podocyte differentiation. In addition, other correlative cells of nephron, such as capillary endothelial cells and parietal epithelial cells, also play crucial roles in kidney development. Further investigation of the miR-200s/RSAD2 signal in these cell types will strengthen our understanding of the miR-200s/RSAD2 relationships.

**Figure 8:**
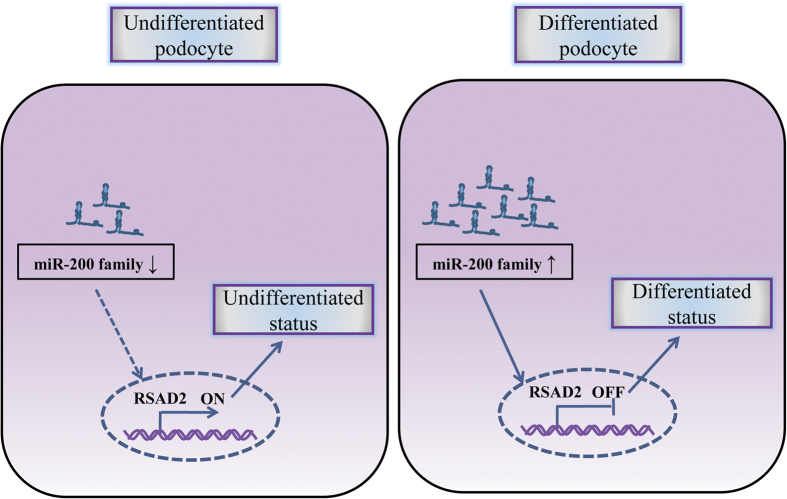
Proposed mechanism of miR-200 family modulating RSAD2 protein and their roles in podocyte differentiation. Down-regulation of miR-200 family fail to inhibit the RSAD2 protein expression in undifferentiated podocytes (*left*). Up-regulation of miR-200 family inhibits the RSAD2 protein expression, the process of which significantly promotes podocyte differentiation (*right*).

In the present study, we demonstrated that miR-200a, miR-200b and miR-429 are significantly up-regulated during the podocyte differentiation. MiR-200 family restraint reveals a significant inhibition in podocyte differentiation. More intriguingly, miR-200 family directly inhibited the RASD2 expression, which can reverse miR-200a/200b/429-mediated promotion of cell differentiation (Fig. 8). It is the first report on a novel role of the antiviral protein RSAD2 as a regulator in cell differentiation. Hence, these findings indicate that miR-200 family may potentially promote podocyte differentiation through repression of RSAD2 expression.

## Materials and Methods

### Cell cultures

Mouse podocytes from a conditionally immortalized cell line (MPC) were cultured, as described previously^35^. Briefly, all cells were grown on flasks coated with type I collagen (Sigma, St. Louis, MO, USA) under growth-permissive conditions at 33 °C in RPMI-1640 medium (Gibco, Gaithersburg, MD, USA) supplemented with 10% fetal bovine serum (Gibco, Gaithersburg, MD, USA), 20 U/ml mouse recombinant interferon-gamma (IFN-γ, Sigma, St. Louis, MO, USA) and 100 U/ml penicillin plus 100 mg/ml streptomycin (Sigma, St. Louis, MO, USA) under humidified atmosphere containing 5% CO2. For podocytes to acquire a differentiated and quiescent phenotype, cells were maintained in nonpermissive conditions at 37 °C in the absence of IFN-γ to inactivate the SV40 T antigen and allow the cells to differentiate (DMPC) for at least 2 weeks.

### Plasmids, miRNA mimics and miRNA inhibitors

Total RNA from differentiated podocytes was extracted with Trizol (Invitrogen, Carlsbad, CA, USA) and reversed with PrimeScript^TM^ RT reagent Kit (Takara Biotechnology, Japan). The whole coding sequences of RSAD2 (NM_021384.4) were generated using a *TaKaRa Ex Taq*^®^ kit (Takara Biotechnology, Japan) using the following primers with the under lined restriction sites *Xho*I and *Bam*HI (forward: 5′-CCGCTCGAGATGGGGATGCTGGTGCC-3′; reverse:5′-CGGGATCCTCA CCAGTCCAGCTTCAGGT-3′), and cloned into pEGFP-N1 vector.

Transient transfection of plasmid, miRNA mimic and miRNA inhibitor were performed with Lipofectamine 2000 Transfection Reagent according to the manufacturer’s (Invitrogen, Carlsbad, CA, USA) instructions. MiRNA mimics and negative control (Dharmacon/GE Healthcare, Mickleton, NJ, USA) were used at a final concentration of 20 nM; miRNA inhibitors (Dharmacon/GE Healthcare, Mickleton, NJ, USA) were used at a final concentration of 100 nM. MicroRNA inhibitor negative control (Dharmacon/GE Healthcare, Mickleton, NJ, USA) were used at a final concentration of 10 nM.

### Dual luciferase reporter assay

3′-UTR complementary DNA (cDNA) fragments of the *RSAD2* genes were amplified from differentiated podocytes by PCR. The 1083 bp fragment of the *RSAD2* 3′-UTR containing the miR-200a/200b/429 targeting sequence (CAGTGTT) was cloned into the psiCHECKTM-2 dual Luciferase reporter plasmid (Promega, Madison, WI, USA) at the 3′ end of the coding sequence of R. reniformis luciferase to produce psiCHECK-WT-*RSAD2* using PCR (primer Forward: 5′ ACCTCTGCC CTAACCTCACC CTC 3′; Reverse: 5′ GTTACCATACATTAGAGCGATGC 3′). To produce the point mutations (psiCHECK-MT-*RSAD2*) of the miR-200a/200b/429 targeting sites, the mutant fragments of *RSAD2* 3′-UTR (miR-200a/200b/429 targeting sites mutated to TGACAGG) were generated using the site-directed Gene Mutagenesis Kit (Beyotime, Haimen, Jiangsu, PR China). For the reporter assays, HEK293 cells were cultured to approximately 80% confluence in a six-well plate and then co-transfected with vectors and miRNA mimics using Lipofectamine 2000 Transfection Reagent (Invitrogen, Carlsbad, CA, USA) and Lipofectamine™ RNAi Max reagent (Invitrogen, Carlsbad, CA, USA), according to the manufacturer’s protocol. After 48 h, the activities of Firefly and Renilla luciferase were measured using the dual-luciferase reporter assay system (Promega, Madison, WI, USA), and Renilla luciferase activity was normalized to the Firefly luciferase activity and are presented as a fold increase relative to the control condition.

### RNA extraction and real-time quantitative PCR (qPCR)

Total RNA isolation and qPCR for detecting miR-200a, miR-200b, miR-429 and RSAD2 mRNA expression were carried out by the following procedures. Briefly, total RNA was extracted using TRIZOL reagent (Invitrogen, Carlsbad, CA, USA) according to manufacturer’s instructions. For miRNAs expression, total RNAs smaller than 200 nt were isolated using the miRcute miRNA Isolation Kit (Tiangen Biotech, Beijing, China). One microgram of total miRNA per sample was copied into complementary DNA (cDNA) using the miRcute miRNA First-strand cDNA Synthesis Kit (Tiangen Biotech, Beijing, China). One microliter of reverse transcription reaction was used for each specific miRNA qPCR assay, which was amplified using miRcute miRNA qPCR Detection Kit (Tiangen Biotech, Beijing, China) following the manufacturer’s instructions. MiRNAs expression levels were normalized to GAPDH level. For gene expression, RNAs were retro-transcribed with the PrimeScript^TM^ RT reagent kit (Takara Biotechnology, Japan) and qPCR performed using SYBR^®^ Premix Ex Taq^TM^ reagent kit (Takara Biotechnology, Japan). Gene expression levels were normalized to GAPDH level. All reactions were done in triplicate using Mastercycler ep realplex 2 (EppendorfAG, Hamburg, Germany) and expression levels calculated using the comparative CT method (2^−ΔΔCT^).

### Oligonucleotide sequences

Sequences of PCR primers and RNA oligonucleotides used for real-time PCR, RSAD2 coding sequence, RSAD2 3′-UTR and RSAD2 3′-UTR site mutagenesis cloning, miR-200a, miR-200b and miR-429 detecting are shown in supplementary data, Table S1.

### Antibodies

Mouse monoclonal antibody to Nestin, Rabbit polyclonal antibody (hAb) to CD2AP, Rabbit polyclonal antibody (hAb) to WT1, Rabbit polyclonal antibody (hAb) to RSAD2 and mouse monoclonal antibody to β-Actin were purchased from Santa Cruz Biotechnology (Santa Cruz, CA). Rabbit polyclonal antibodies (hAb) to Nephrin were purchased from Abcam (Abcam Inc., Cambridge, MA). The secondary peroxidase-conjugated antibodies were from Promega (Madison, WI). Secondary antibodies coupled with green-fluorescent Alexa-488 and red-fluorescent Alexa-647 were from Invitrogen (Invitrogen, Carlsbad, CA).

### Western blot analysis

Preparation of cell lysates has been described in our previous studies. Equal amounts protein loadings were separated by SDS-PAGE on 8% or 10% gels and electrophoretically transferred to a PVDF membrane. Nonspecific binding sites were blocked with 5% nonfat milk powder in phosphate-buffered saline (PBS) and 0.05% Tween 20 for 1 h at room temperature. Membranes were incubated in the following primary antibodies at appropriate concentrations: RSAD2 (1:1000 dilution), β-actin (1:1500 dilution), Nestin (1:1000 dilution), Nephrin (1:1000 dilution), CD2AP (1:1000 dilution) and WT1 (1:1000 dilution). Secondary antibodies were used at 1:2500. Following additional washes before application of chemiluminescent substrate (Millipore, Billerica, MA, USA), the membranes were exposed on MXG film (Kodak, Rochester, NY) using varying exposure times. β-actin was used as an internal control. The quantification of bands was performed using the Image J software (NIH, Bethesda, MD).

### Fluorescent staining

Cells cultured on glass coverslips were fixed with 4% paraformaldehyde in PBS for 15 min at 4 °C, and incubated in blocking buffer (10% normal goat serum and 0.3% Triton X-100) for 30 min at 37 °C. Cells were labeled with Nephrin hAb (1:200 dilution) diluted in 5% of normal goat serum, 0.3% Triton X-100 in PBS for 2 h at 37 °C. After three times of gentle wash with PBS, Secondary antibodies coupled with green-fluorescent Alexa-488 were used at 1:500. For the observation of cytoskeletal assembly, cells were stained for 30 min in PBS containing 100 ng/ml rhodamine phalloidin (Cytoskeleton, Inc) and 5 μg/ml DAPI (Sigma, St. Louis, MO, USA), rinsed in PBS for three times, and observed under a confocal laser scanning fluorescent microscope at FV1000 (Olympus, Tokyo, Japan). Images were then processed by Photoshop 7.0 (Adobe, San Jose, CA).

The tissue samples were perfusion-fixed with 4% paraformaldehyde in 0.1 M phosphate buffer (pH 7.4), sliced in 4 mm thick sections and incubated in blocking buffer (10% normal goat serum and 0.3% Triton X-100) for 30 min at 37 °C. Sections were labeled with RSAD2 hAb (1:200 dilution) diluted in 5% of normal goat serum, 0.3% Triton X-100 in PBS for 2 h at 37 °C. After three times of gentle wash with PBS, Secondary antibodies coupled with red-fluorescent Alexa-647 were used at 1:500. For the observation of nuclear, cells were stained for 2 min in PBS containing 5 μg/ml DAPI (Sigma, St. Louis, MO, USA), rinsed in PBS for three times, and observed under a confocal laser scanning fluorescent microscope at FV1000 (Olympus, Tokyo, Japan). Images were then processed by Photoshop 7.0 (Adobe, San Jose, CA).

### Annexin V/propidium iodide (PI) staining assay

To determine type and ratio of apoptotic and necrotic cell death, cells were detected with AnnexinV-FITC/PI Detection Kit (Tianjin Sungene Biotech, China) according to the manufacturer’s protocol. Briefly, cells were harvested, washed in phosphate-buffered saline (PBS) and stained for 15 min at room temperature, with Annexin V-FITC (5 μl) and PI (5 μl) in binding buffer in the dark. The cells were then analyzed in a FACSCalibur flow cytometer (Beckman-Coulter, Fullerton, CA) equipped with CellQuest software (BD Biosciences, San Jose, CA).

### TUNEL staining

TUNEL staining was conducted using the *in situ* colorimetric TUNEL apoptosis assay kit (Beyotime, China), according to the manufacturer’s manual. Briefly, the sections were washed one time in PBS and incubated in 4% paraformaldehyde in 0.1 M phosphate buffer (pH 7.4) for 30 min. The sections were washed one time with PBS. Then sections were incubated in the cold 0.1% Triton X-100 for 2 min. The sections were washed two times with PBS. Then the sections were incubated with TdT enzyme solution for 60 min at 37 °C in darkness. Then sections were rinsed in PBS for three times, and observed under a confocal laser scanning fluorescent microscope at FV1000 (Olympus, Tokyo, Japan). Images were then processed by Photoshop 7.0 (Adobe, San Jose, CA).

### Cell proliferation assay

The status of mouse podocyte viability was assessed using the colorimetric reagent, tetrazolium salt 2-(2-methoxy-4-nitrophenyl)-3-(4-nitrophenyl)-5-(2,4-disulfophenyl)-2H- tetrazolium monosodium salt (WST-8), commercially available as Cell Counting Kit-8 (CCK-8) reagent (Beyotime Inst Biotech, China). Briefly, cells were seeded in 96-well plates with 1 × 10^4^ cells in 200 μl/well and cultured for 48 h. After that, cultures were incubated with CCK-8 (20 μl) for 1 h at 37 °C. At last, the absorbance was assessed on a microplate reader (Multiskan Mk3, Thermo Labsystems, Helsinki, Finland).

### Cell cycle analysis

Cells were harvested by centrifugation at 1500 rpm for 5 min. Supernatants were discarded and cells were suspended in ice-cold PBS and fixed in 70% ice-cold ethanol. Upon analysis, the cells were washed with PBS and then resuspended in DNase-free RNase A (100 μg/ml) and left at 37 °C for 30 min. Cells were then incubated in PBS containing propidium iodide (10 μg/ml) for 30 min at 4 °C. The cell cycle distribution was measured by a flow cytometer (BD FACS Calibur) equipped with CellQuest software.

### Statistical analysis

All data were expressed as mean ± SD. Independent-samples t-tests were applied where two groups of data were compared. The mean values are presented in bar charts, with T-bars referring to standard deviations. Multiple samples were statistically analysed for significance using one-way analysis of variances (One-Way ANOVA). Post hoc statistics were obtained using the LSD test (Fisher**’s**least significant difference t-test) for multiple comparisons. All of the statistical analyses were performed using SPSS 11.5 software (IBM, Chicago, IL). *P*-values less than or equal to 0.05 were considered to be significant. All experiments were performed at least in triplicate.

## Additional Information

**How to cite this article**: Li, Z. *et al*. miR-200 family promotes podocyte differentiation through repression of RSAD2. *Sci. Rep.* **6**, 27105; doi: 10.1038/srep27105 (2016).

## Supplementary Material

Supplementary Information
