# Supplementary material for: miR-200 family promotes podocyte differentiation through repression of RSAD2
Source: Sci Rep. 2016 Jun 2;6:27105. doi: 10.1038/srep27105 (PMC4890021; doi:10.1038/srep27105)
Supplement: Supplementary Information [file srep27105-s1.pdf]

miR-200 family promotes podocyte differentiation through repression of RSAD2

Li Zhigui, Yin Hongqiang, Hao Shuang, Wang Lifeng, Gao Jing, Tan Xiaoyue, Yang Zhuo*

College of Medicine, State Key Laboratory of Medicinal Chemical Biology, Tianjin Key Laboratory of Animal Models and Degenerative Neurological Diseases, Nankai University, Tianjin300071, China

*Correspondence for Proofs:

Professor Yang Zhuo, College of Medicine, Nankai University, Tianjin, 300071, China. Tel: 86-22-23504364. Fax: 86-22-23502554. E-mail: zhuoyang@nankai.edu.cn

Table S1 Oligonucleotides used in qPCR and cloning studies

Primer Name	Sequence (5'to 3')
**qPCR**	
RSAD2 forward	CCGCTCGAGATGGGGATGCTGGTGCC
RSAD2 reverse	CGGGATCCTCACCAGTCCAGCTTCAGGT
miR-200a	TAACACTGTCTGGTAACGATGT
miR-200b	TAATACTGCCTGGTAATGATGA
miR-429	TAATACTGTCTGGTAATGCCGT
miR-200c	TAATACTGCCGGGTAATGATGGA
miR-141	TAACACTGTCTGGTAAAGATGG
GAPDH forward	AGGTCGGTGTGAACGGATTTG
GAPDH reverse	TGTAGACCATGTAGTTGAGGTCA
**RSAD2 3'UTR**	
RSAD2 forward	ACCTCTGCCCTAACCTCACCCTC
RSAD2 reverse	GTTACCATACATTAGAGCGATGC
**RSAD2 3'UTR mutagenesis**	
RSAD2 forward	CATTGGTTACATTTT**TGACAGG**AAATCTGGGTGC
RSAD2 reverse	GTAACCAATGTAAAA**ACTGTCC**TTTAGACCCACG
**RSAD2 cloning**	
RSAD2 forward	CCGCTCGAGATGGGGATGCTGGTGCC
RSAD2 reverse	CGGGATCCTCACCAGTCCAGCTTCAGGT

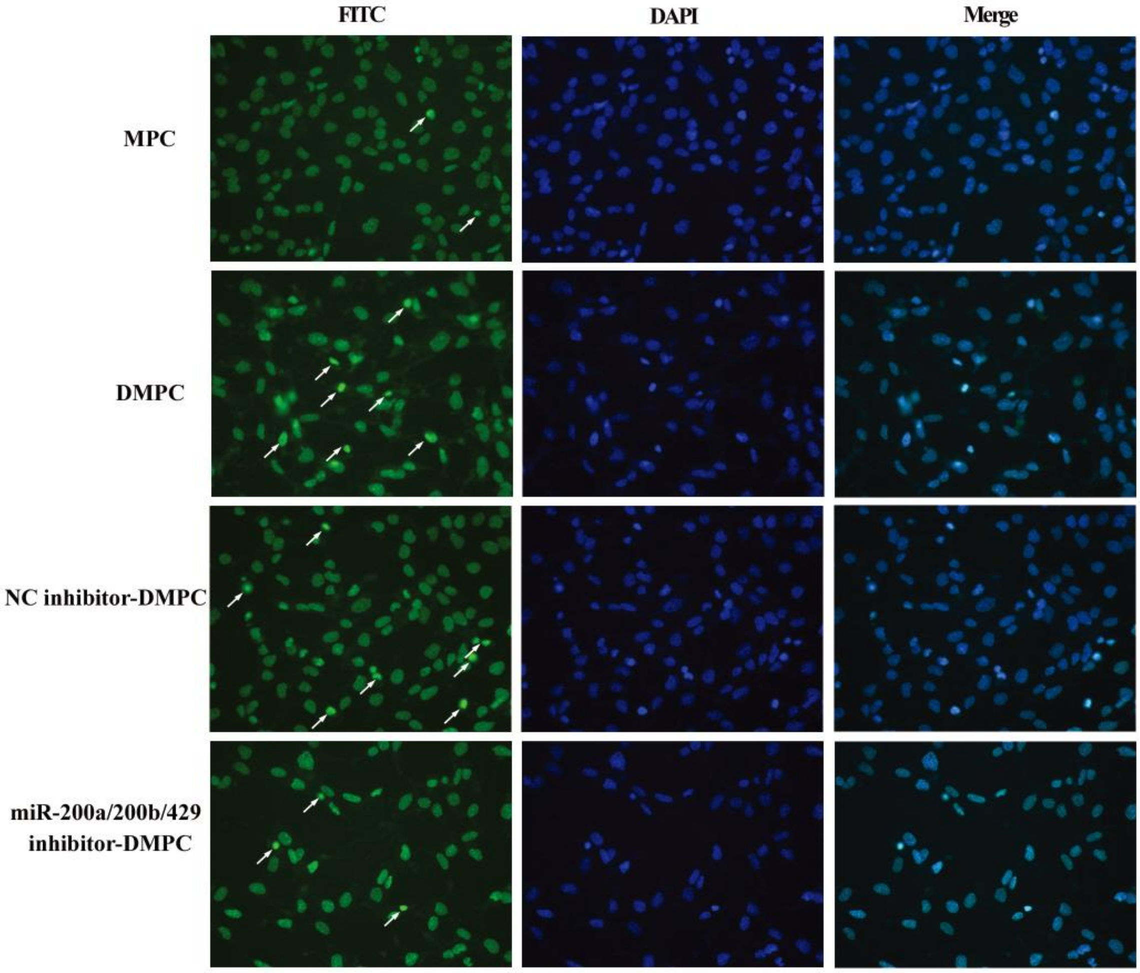

Fig. S1 The morphological validation of cell apoptosis by TUNEL assay. Apoptotic cell DNA were labeled with FITC (green). Podocyte nuclears were also stained with DAPI (blue).

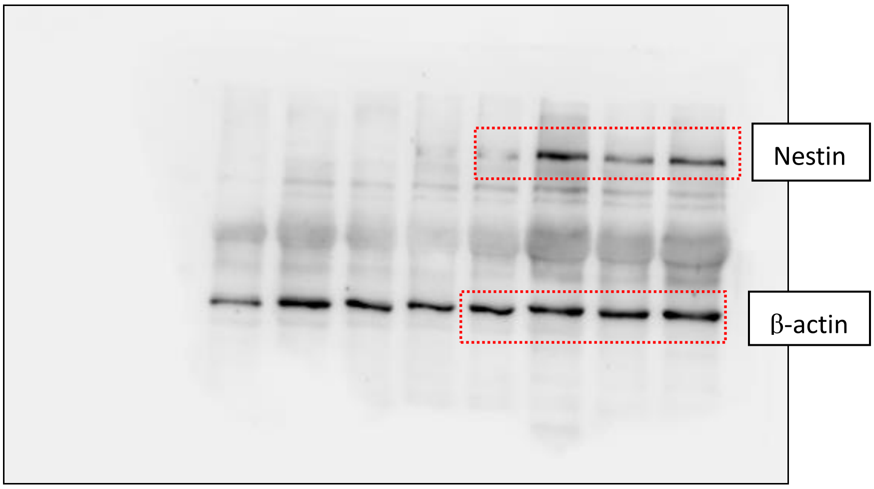

Fig. S2 The corresponding full-length blot of Nestin and β-actin was shown above.

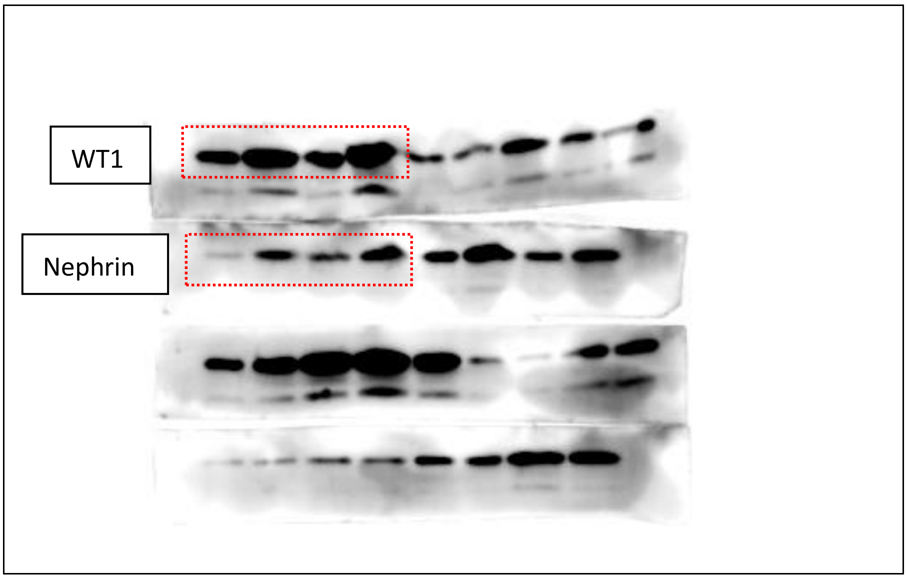

Fig. S3 The corresponding full-length blot of WT1 and Nephrin was shown above.

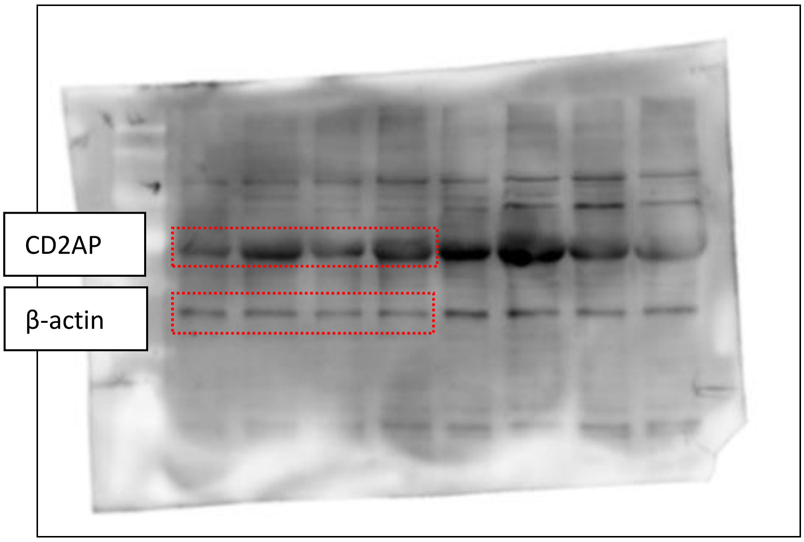

Fig. S4 The corresponding full-length blot of CD2AP was shown above.

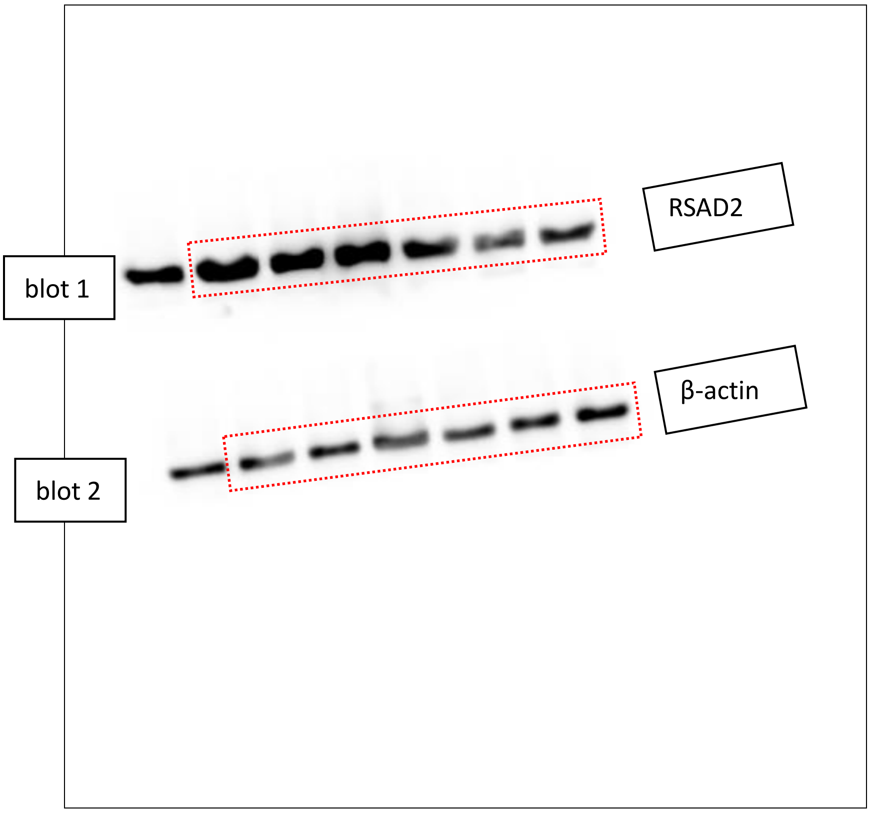

Fig. S5 The corresponding full-length blot of RSAD2 and β-actin was shown above.

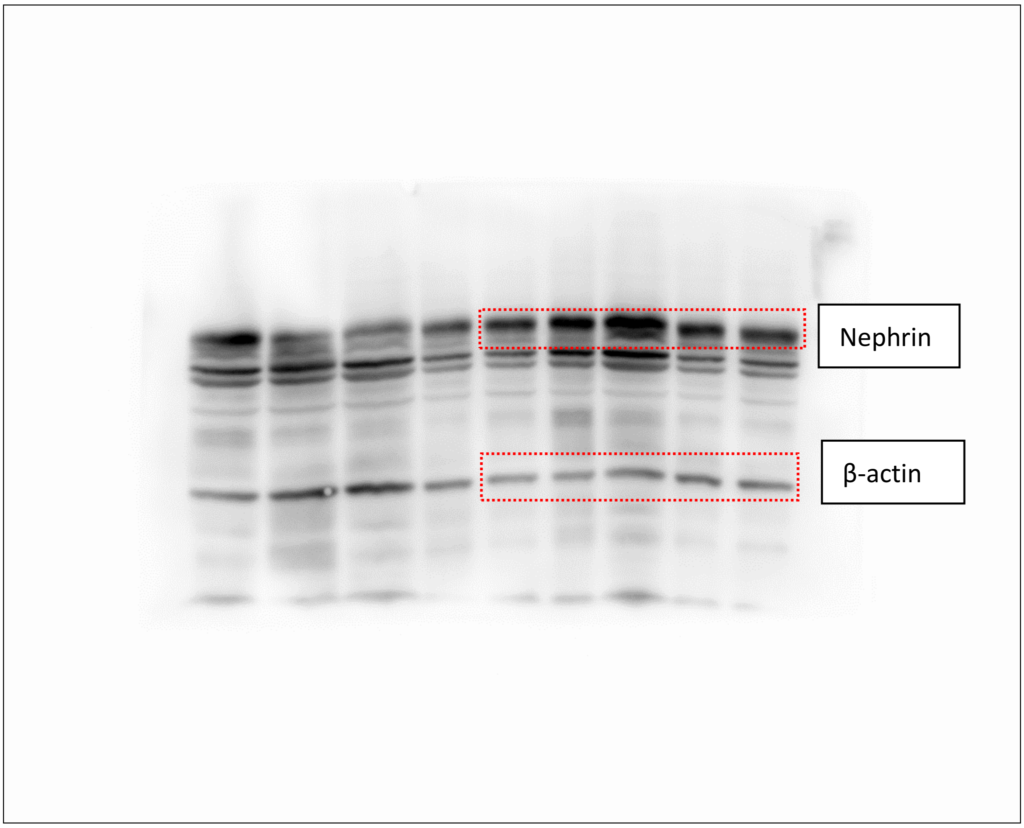

Fig. S6 The corresponding full-length blot of Nephrin and β-actin was shown above.

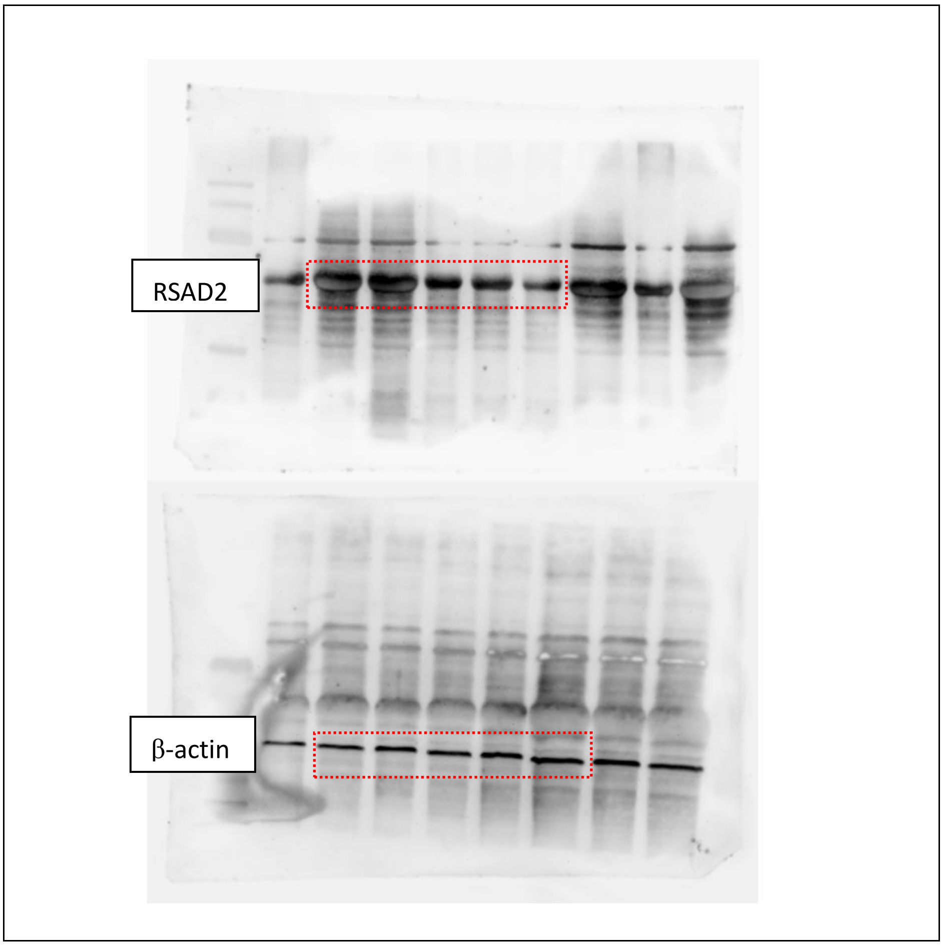

Fig. S7 The corresponding full-length blot of RSAD2 and β-actin was shown above.
